# Seasonality and Prevalence of *Leishmania major* Infection in *Phlebotomus duboscqi* Neveu-Lemaire from Two Neighboring Villages in Central Mali

**DOI:** 10.1371/journal.pntd.0001139

**Published:** 2011-05-10

**Authors:** Jennifer M. Anderson, Sibiry Samake, Giovanna Jaramillo-Gutierrez, Ibrahim Sissoko, Cheick A. Coulibaly, Bourama Traoré, Constance Soucko, Boubacar Guindo, Dansine Diarra, Michael P. Fay, Phillip G. Lawyer, Seydou Doumbia, Jesus G. Valenzuela, Shaden Kamhawi

**Affiliations:** 1 Laboratory of Malaria and Vector Research, National Institutes of Allergy and Infectious Diseases, National Institutes of Health, Rockville, Maryland, United States of America; 2 Faculty of Medicine, Pharmacy and Odontostomatology, University of Bamako, Bamako, Mali; 3 Faculty of Science and Technology, University of Bamako, Bamako, Mali; 4 Biostatistics Research Branch, National Institutes of Allergy and Infectious Diseases, National Institutes of Health, Rockville, Maryland, United States of America; 5 Intracellular Parasite Biology Section, Laboratory of Parasitic Diseases, National Institutes of Allergy and Infectious Diseases, National Institutes of Health, Rockville, Maryland, United States of America; Lancaster University, United Kingdom

## Abstract

*Phlebotomus duboscqi* is the principle vector of *Leishmania major*, the causative agent of cutaneous leishmaniasis (CL), in West Africa and is the suspected vector in Mali. Although found throughout the country the seasonality and infection prevalence of *P. duboscqi* has not been established in Mali. We conducted a three year study in two neighboring villages, Kemena and Sougoula, in Central Mali, an area with a leishmanin skin test positivity of up to 45%. During the first year, we evaluated the overall diversity of sand flies. Of 18,595 flies collected, 12,952 (69%) belonged to 12 species of *Sergentomyia* and 5,643 (31%) to two species of the genus *Phlebotomus*, *P. duboscqi* and *P. rodhaini*. Of those, *P. duboscqi* was the most abundant, representing 99% of the collected *Phlebotomus* species. *P. duboscqi* was the primary sand fly collected inside dwellings, mostly by resting site collection. The seasonality and infection prevalence of *P. duboscqi* was monitored over two consecutive years. *P. dubsocqi* were collected throughout the year. Using a quasi-Poisson model we observed a significant annual (year 1 to year 2), seasonal (monthly) and village effect (Kemena versus Sougoula) on the number of collected *P. duboscqi*. The significant seasonal effect of the quasi-Poisson model reflects two seasonal collection peaks in May-July and October-November. The infection status of pooled *P. duboscqi* females was determined by PCR. The infection prevalence of pooled females, estimated using the maximum likelihood estimate of prevalence, was 2.7% in Kemena and Sougoula. Based on the PCR product size, *L. major* was identified as the only species found in flies from the two villages. This was confirmed by sequence alignment of a subset of PCR products from infected flies to known *Leishmania* species, incriminating *P. duboscqi* as the vector of CL in Mali.

## Author Summary

Female sand flies transmit a parasite called *Leishmania* that causes a disease called cutaneous leishmaniasis (CL). Several species of sand flies are found in West Africa, but only one species, *Phlebotomus duboscqi*, has been proven to transmit the parasite. Cutaneous Leishmaniasis has also been reported from Mali, Central West Africa, but the sand fly transmitting the parasite and its annual abundance has not been established, until now. Sand flies were collected during three consecutive years from two neighboring villages in Central Mali, Kemena and Sougoula, where CL is present. *P. duboscqi* was collected year-round and was the dominant sand fly inside of and surrounding human dwellings. Other sand fly species, known not to be vectors of CL, were primarily found outside the village. Additionally, *P. duboscqi* females were found infected with *L. major,* the same *Leishmania* species identified from human CL cases in Mali. The estimated infection prevalence of *P. duboscqi* females was 2.7%. Interestingly, the sand fly abundance and infection prevalence was similar in the two villages despite a previous report indicating a disparate *L. major* exposure rate in humans. This study greatly enhances our knowledge of CL transmission in Mali, poorly studied in this country to date.

## Introduction

In West Africa *Phlebotomus duboscqi* Neveu-Lemaire is the most important vector of *Leishmania major*, the causative agent of cutaneous leishmaniasis (CL) [1], [2]. *P. duboscqi* has been incriminated as the vector of *L. major* in Senegal [3] and suspected as the vector of CL in Burkina Faso [4], Niger [5], [6], The Gambia [7], Ghana [8], Cameroon [9] and Mali [10], [11], [12]. The first report of *P. duboscqi* in Mali was from Hombori in 1906 [13] with additional reports from Timbuctu in 1913 [14] and from Bamako and Nioro in 1943 [10]. Later work by Lariviere [11] and Desjeux [1] found *P. duboscqi* in all regions of the country.

Cutaneous Leishmaniasis is endemic in Mali with cases historically occurring in the districts of Nioro and Segou [11], [15]. The first published report of CL in Mali concerned two cases identified from Nioro in 1944 [16]. Later studies reported leishmanin skin test positivity rates between 10 and 61%, suggesting that *Leishmania* is endemic in Mali [15], [17], [18], [19]. *Leishmania major* was first identified as the causative agent of CL in Mali by isoenzyme analysis of parasites isolated from skin samples taken from a lesion of a tourist visiting Mopti [20] and a local resident living in the same region [21].

Despite the identification of *L. major* as the causative agent of CL in Mali, and although suspected as the vector, no one has identified the parasite in *P. duboscqi*. Here, we report on a three year survey to evaluate the diversity of sand flies and the seasonal abundance of *P. duboscqi* in Kemena and Sougoula, two villages endemic for CL in the District of Baroueli, Region of Segou, in Central Mali. Furthermore, we report for the first time the detection and annual prevalence of *L. major* parasites in *P. duboscqi* sand flies collected from the study sites.

## Methods

### Site description

Sand flies were collected from two neighboring villages, Kemena (12°33′ N–6°33′ W) and Sougoula (13°05′ N, –6°53′ W), in the Baroueli Health District, Region of Segou, Mali. Both villages have a population size of approximately 1000 inhabitants. Each village is organized into a labyrinth of adjoining compounds within which a single extended family resides in several sleeping, cooking, and storage houses. Houses are constructed of clay bricks plastered with mud and straw, and with thatched or metal roofs. Domestic animals, such as goats, sheep, and chickens are kept within the confines of a family compound while cows are maintained in corrals located around the perimeter of the village. Both villages have a limited infrastructure and lack electricity and running water. The climate consists of three distinct seasons: a dry season from March to June (temperature range 27–40°C; monthly average rainfall 5.2 mm), a rainy season from June to September (temperature range 25–35°C, monthly average rainfall 82.42 mm), and a third temperate season from October to February (temperature range 20–35°C; monthly average rainfall 3.3 mm). Vegetation is sparse and is characterized by the presence of sporadically placed trees such as shea (*Vitellaria paradoxa)*, acacia (*Faidherbia albida*) and neem (*Azadirachta indica*) and small bushes. Most of the land surrounding each village is dedicated for agricultural use.

### Sand fly collection

Sand flies were collected using 1) dark activated, CDC miniature light traps fitted with double ring fine mesh collection bags (John W. Hock Company, Gainesville, FL), 2) sticky traps consisting of single sheets of A4 paper (21×29.5 cm) coated on both sides with castor oil and mounted vertically on pegs, onto which randomly impinging sand flies would adhere (used for the sand fly diversity study only), and 3) mouth aspirators (John W. Hock Company, Gainesville, FL) for collection of resting flies inside of houses used for sleeping. All sand flies were sorted by sex, species and blood meal status, and placed in tubes containing silica gel and cotton until processed. Minimum and maximum temperatures, rainfall and relative humidity for the months of July 2006 to June 2008 were collected from the nearest available weather station in Segou, Mali. Oral informed consent was obtained from head of households for indoor collection of sand flies. Households where consent was given were listed in a written log kept by the entomological team for reference.

#### Sand fly diversity

To assess diversity, sand flies were collected monthly from March 2005 through June 2006 using all three collection methods. Specifically, five light traps were placed inside houses used for sleeping and two light traps were placed outside houses within the same compound in five different compounds for three consecutive nights per village (n = 2; Kemena and Sougoula) for a total of 210 trap-nights per month. Light traps were placed at dusk and collected at dawn the next day. One sticky trap was placed in each of 10 sleeping houses scattered around the village and 10 sticky traps were placed in natural holes found in trees along the perimeter of each village for three nights per month, per village. Sand flies were removed from sticky traps using soapy water with a fine-haired paint brush or dissecting needle and placed in soapy water to remove the castor oil, rinsed in pure water and transferred to vials. Resting site collections were conducted by three people, each working in one of three rooms within a compound for 20 and 15 minutes at dawn and dusk, respectively, in three compounds on two consecutive days for a monthly total of 70 min per person, per village. All sand flies collected were stored in 70% ethanol until processed.

#### Seasonality and Infection prevalence of *P. duboscqi*

**Figure 1:**
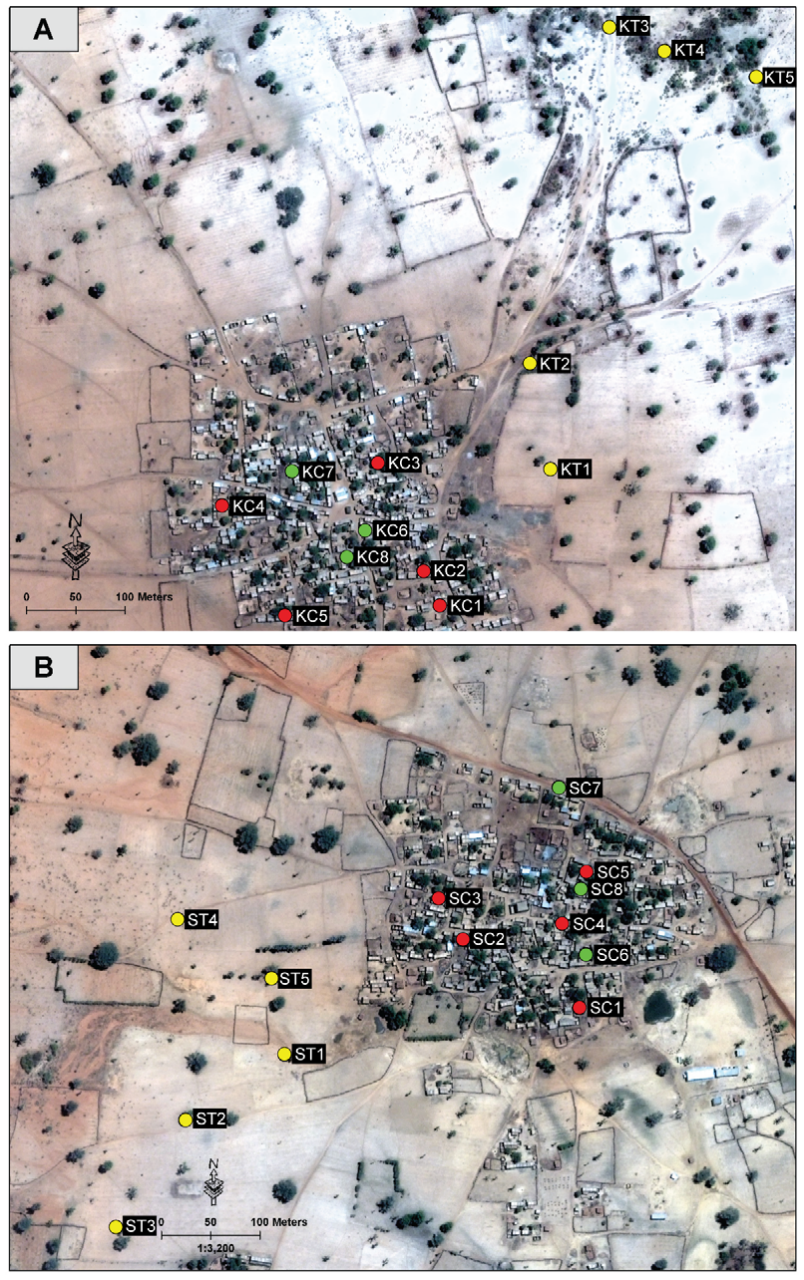
Satellite image of study villages in Central Mali. Satellite image of the two study villages, Kemena (A) and Sougoula (B) illustrating the sites of monthly sand fly collections (circles) during the study of *Phlebotomus duboscqi* seasonality, July 2006 to June 2008. Red  =  compounds of indoor/outdoor house light trap collections; green  =  compounds of resting site collections; yellow  =  light trap location near trees. Image collected on May 22, 2006 by the Quickbird Satellite (DigitalGlobe, Inc. Longmont, CO USA).

To assess the seasonality and infection prevalence of *P. duboscqi*, light trap and resting site collections were conducted in each village on two consecutive nights per month from July 2006 through June 2008. A total of 25 light traps were placed in and around each village for a total of 100 trap-nights per month. Light traps were placed inside and outside 20 houses within five compounds for a total of four light traps per compound (Figure 1). Additionally, five light traps were placed near trees located outside each village. Resting site collections using mouth aspirators were conducted for 20 mins in two houses in each of three different compounds (six houses total) per village (Figure 1) for a total of four person hours each month (two hours at dawn and two hours at dusk) per village. *Phlebotomus* sand flies were grouped based on village, collection month, collection technique, location, genera and sex and were stored dry in tubes containing silica desiccant until processed. *Phlebotomus* females collected from individual light traps or resting sites were further separated into blood fed and non-blood fed pools of no more than 20 sand flies. *Sergentomyia* species were archived in 70% ethanol.

### Species identification

The head and terminal segments of the abdomen containing the genitalia of each sand fly were carefully removed and placed into 96-well plates containing a solution of lacto-phenol clearing solution (Bioquip). After 24 h incubation at room temperature, the head and terminalia were fixed onto a glass slide, examined using a light microscope and identified using dichotomus keys [22].

### Detection of *Leishmania* major

From June 2006-July 2008, the abdomens of blood fed and non-blood fed *Phlebotomus* females from the same collection location were grouped in pools of no more than 20 individuals and placed in a microfuge tube containing lysis buffer (5.84 g/L NaCl, 68.5 g/L Sucrose, 12.10 g/L Tris, pH 9.1, 100 ml EDTA 0.5 M solution and 50 ml 10% SDS solution). After incubating overnight at 4°C the tissue was macerated using a pestle for 2 min then incubated for 30 min at 65°C. After the addition of 10 µl cold potassium acetate the samples were incubated for 30 min at 4°C and then centrifuged for 10 min at 14,000 RPM. The DNA was precipitated using 70% ethanol and resuspended in 100 µl water. The DNA concentration of each extraction was determined using a NanoDrop (Thermo Scientific Inc., Wilmington, DE). Samples with less than 4 ng/µl of DNA were removed from the sample set. *Leishmania* DNA was detected by PCR using forward and reverse primers for *Leishmania* sp. (Uni21/Lmj4) as described in [23]. PCR Primers targeting the sand fly tubulin gene were used as a control for template fidelity (PpTub-P24F 5′-GCG ATG ACT CCT TCA ACA C and PpTub-P24R 5′-TCA GCC AGC TTG CGA ATA C) [24].

A representation of PCR products was confirmed by DNA sequencing. Due to difficulties with direct sequencing of the PCR products using the Uni21 and Lmj4 primers, gel-purified PCR products were cloned into the pCR4-TOPO vector using the TOPO TA Cloning Kit for Sequencing (Invitrogen, Carlsbad CA) following the manufacturer's instructions. The clones were sequenced directly using the M13 forward and M13 reverse primers. Resulting sequences were analyzed using DNASTAR sequence analysis software (DNASTAR, Inc., Madison WI). Sequences were compared to published sequences of kDNA from *L. major* (Genbank Accession J04654), *L. infantum* (AF188701), *L. tropica* (Z32841), and *L. donavani* (AF167718) using BLAST (http://blast.ncbi.nlm.nih.gov/), aligned to known *Leishmania* minicircle kinetoplastic DNA using Clustal [25] and edited using BioEdit (http://www.mbio.ncsu.edu/BioEdit/page2.html).

### Statistical analysis

To estimate the prevalence of infection in pooled samples of *P. duboscqi* females, we used the maximum likelihood estimate (MLE) of prevalence accounting for pooling with the confidence interval (CI) estimated by exact methods if the number of unique pool sizes was less than or equal to 3 [26], or otherwise by the skewness-corrected score confidence interval [27]; the estimates and both CIs were calculated using the binGroup R package [28].

To model the sand fly counts or infection rates we used a quasi-Poisson model and tested for significant effects using analysis of deviance and F test [29]. To test for seasonal effects, we tested the overall effect of months after controlling for previous counts and year. In testing for weather effects, we compared models with previous counts, year and months and tested to see if models that additionally added the previous month weather variables (including 4 weather variables at a time; selecting only one [minimum or maximum] of temperature or wild velocity variables) significantly improved the fit. For the models of rates, we estimated the number of infected flies of those tested by the MLE of prevalence and used those counts as responses in the quasi-Poisson model with an offset based on the number of flies tested so that the inferences describe effects on the rates [29]. The quasi-Poisson models were performed using R version 2.12 [30]. Graphs were made using GraphPad Prism 5 (Graphpad Software, California, USA).

## Results

### Sand fly species diversity

From March 2005 to June 2006, 18,595 sand flies were collected in the two villages (9,887 in Kemena and 8,708 in Sougoula) using all three collection methods. Approximately equal numbers of male and female sand flies were collected (9,221 M, 9,374 F). Sixty-nine percent (n = 12,952) of sand flies were identified as one of 12 species in the genus *Sergentomyia*, none of which have been implicated in the transmission of *L. major* (Table 1).

**Table 1:** Sand fly species diversity in two neighboring villages, Central Mali, March 2005-June 2006.

Species	Males	Female	Total	Percent Total
***Phlebotomus***				**30.34**
*P. duboscqi*	3028	2574	5602	99.28
*P. rodhaini*	15	26	41	0.72
***Sergentomyia***				**69.66**
*S. schwetzi*	3684	2444	6128	47.31
*S. antennata*	1797	1627	3424	26.44
*S. dubia*	30	1549	1579	12.19
*S. clydei*	453	570	1023	7.90
*S. africana*	187	228	415	3.20
*S. squamipleuris*	10	208	218	1.68
*S. affinis vorax*	10	63	73	0.56
*S. bedordi*	3	60	63	0.49
*S. fallax*	0	3	3	0.02
*S. buxtoni*	0	17	17	0.13
*S. darlingi*	3	5	8	0.06
*S. christopheri*	1	0	1	0.01
**Total**	9221	9374	18595	100%

Of the *Sergentomyia*, *Sergentomyia schwetzi* Adler, Theodor and Parrot represented the majority with 47.3% of collected specimens, while *Sergentomyia antennata* Newstead was the second most abundant at 26.4%. Ten additional *Sergentomyia* species were collected: *Sergentomyia dubia* Parrot, Mornet, and Cadenat (12.2%), *Sergentomyia clydei* Sinton (7.9%), *Sergentomyia africana* Newstead (3.2%), *Sergentomyia squamipleuris* Newstead (1.7%), *Sergentomyia affinis vorax* Parrot (0.56%), *Sergentomyia bedfordi* Newstead (0.49%), *Sergentomyia fallax* Parrot (0.02%), *Sergentomyia buxtoni* Theodor (0.13%), *Sergentomyia darlingi* Lewis and Kirk (0.06%), and *Sergentomyia christophersi* Sinton (0.01%). The remaining 30% of sand flies collected was identified as one of two species of *Phlebotomus*, the overwhelming majority of which was *P. duboscqi* (n = 5,643, 99.3%). Only 41 *Phlebotomus rodhaini* Parrot (0.7%) were collected (Table 1).

Sticky traps and light traps collected sand flies in about equal numbers (n = 8,290 vs. 8,394), yet the majority of *Sergentomyia* (n = 7,728, 60%) were collected using sticky traps whereas only 10% of *Phlebotomus* (n = 562) were collected using this method. The majority of *Phlebotomus* (n = 3,380, 60%) were collected using light traps. Thirty percent of *Phlebotomus* (n = 1,701) were collected by resting site collection compared to only 1.62% (n = 210) of *Sergentomyia*. Comparing sticky trap and light trap collections from inside and outside houses,the majority of *Phlebotomus* (92%, n = 3,641) were collected inside dwellings whereas the majority of *Sergentomyia* (71%, n = 9,043) were collected outside dwellings.

### Seasonal distribution of *P. duboscqi*

**Figure 2:**
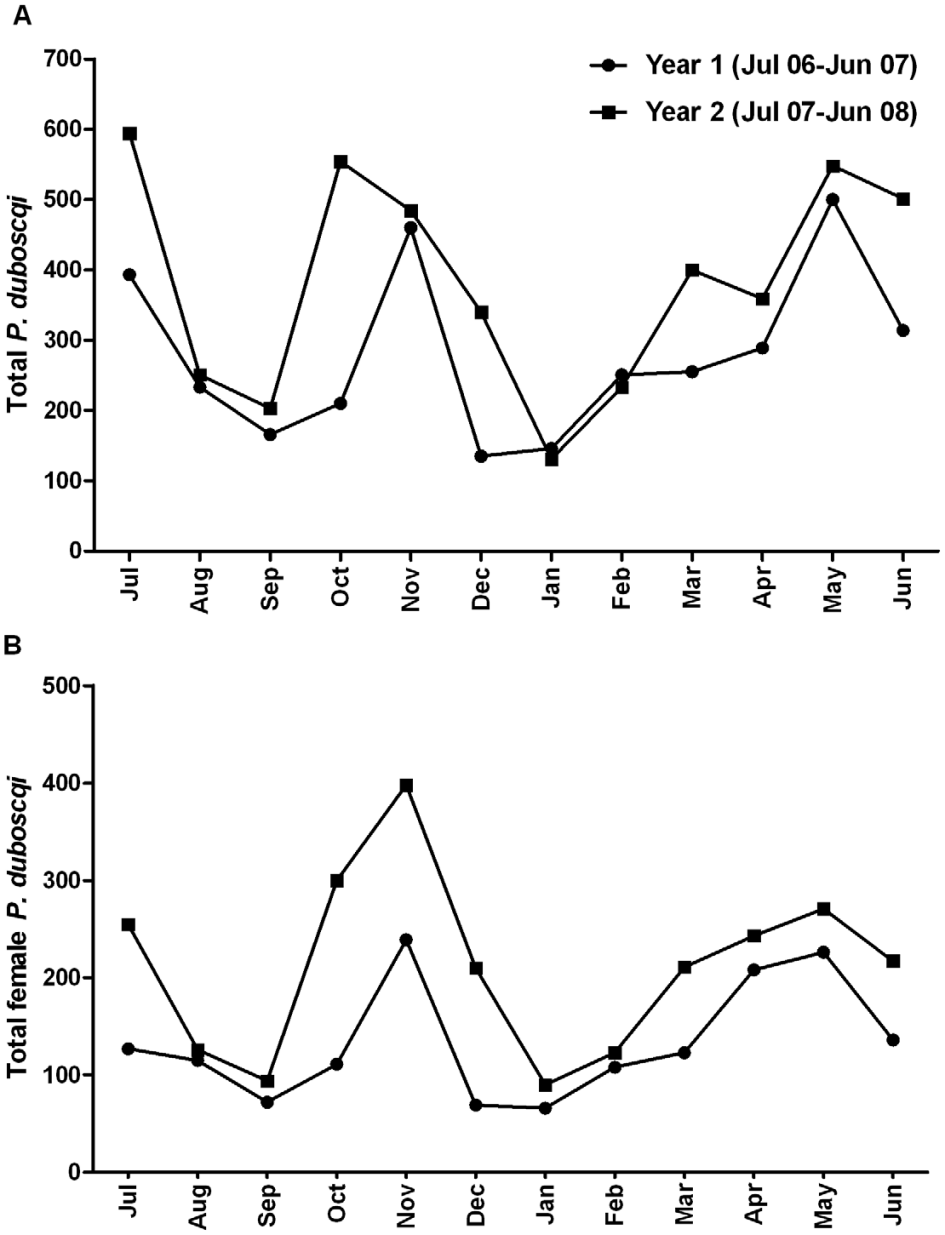
Monthly collections of *Phlebotomus duboscqi* by year in two neighboring villages in Central Mali. The combined total number of *P. duboscqi* (A) and female *P. duboscqi* (B) collected using two collection methods (light trap and resting site collection) during two collection nights per month per village over two consecutive years in the neighboring villages of Kemena and Sougoula in Central Mali.

**Figure 3:**
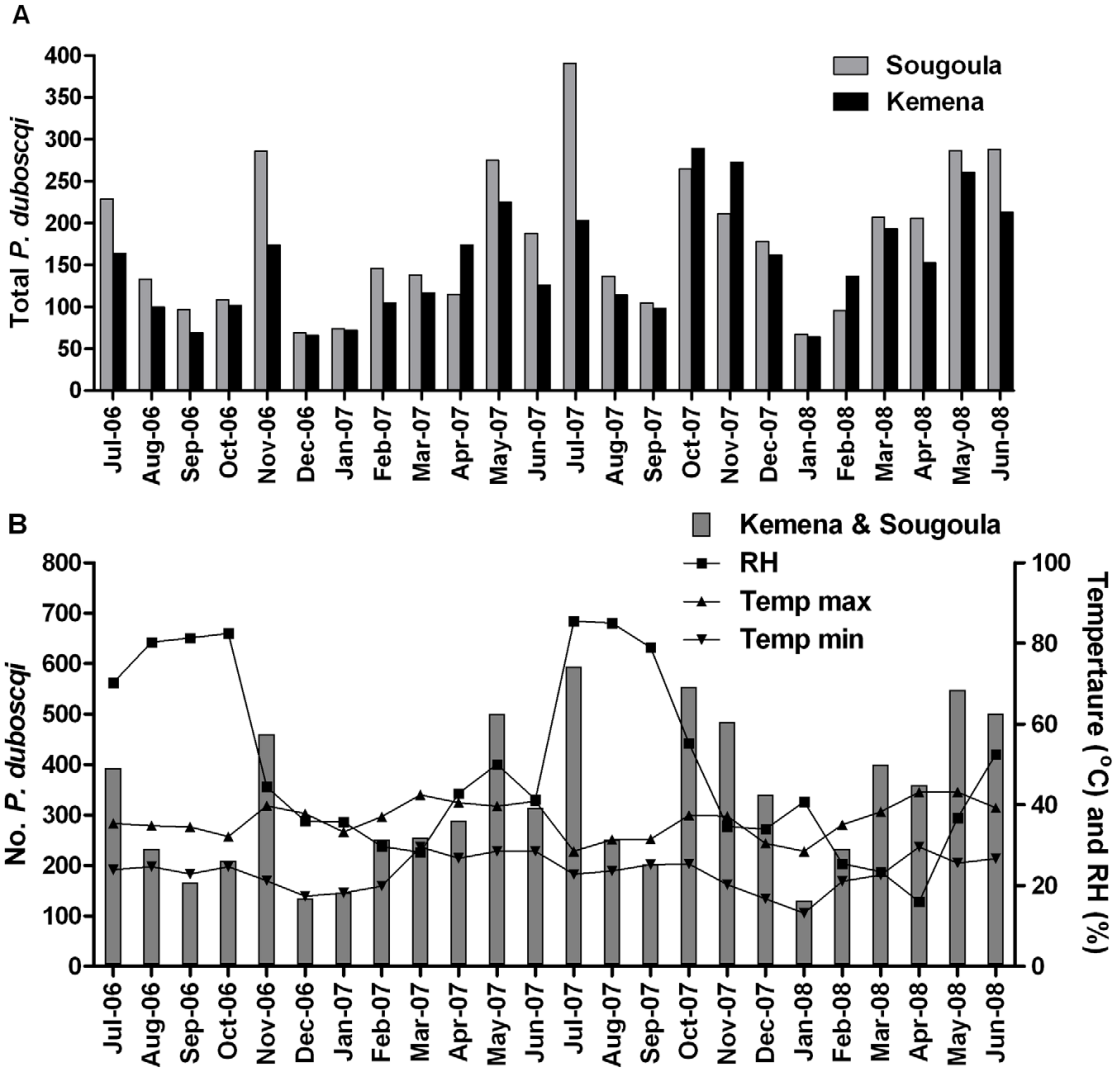
Monthly collections of *Phlebotomus duboscqi* by village and climatic conditions in two neighboring villages in Central Mali. The number of *P. duboscqi* collected over two consecutive years using two methods (light trap and resting site collection) during two collection nights per month in each of the two neighboring villages of Kemena and Sougoula in Central Mali. (A) Comparison of the monthly collections of female *P. duboscqi* in Kemena and Sougoula. (B) Correlation of the main climatic conditions with the monthly collections of *P. duboscqi* from both villages. RH  =  monthly average relative humidity (%). Temp max  =  monthly average daily maximum temperature. Temp min  =  monthly average daily minimum temperature.

From July 2006 to June 2008, 7,950 *P. duboscqi* (3,998 female) were collected. Additionally, a total of 25 *P. rodhaini* were collected during the two years, 17 of which were collected during one month in Kemena (October 2006). Comparing the total number of *P. duboscqi* collected during year one (July 2006–June 2007) and year 2 (July 2006–June 2008), we found that 1.42 times more sand flies were collected in year 2 (p-value 0.0002, 95% CI: 1.19–1.69) (Figure 2A). We observed a similar effect when comparing the annual collections of female *P. duboscqi* (p-value 0.0003) (Figure 2B). Using the quasi-Poisson model, controlling for year and previous count, we observed a significant seasonal effect reflecting the month to month variation in the total number of sand flies collected (p-value <0.0001). A similar effect was observed when we considered only female sand flies (P-value <0.0001). Monthly collection trends were similar during both collection years. We modeled sand fly counts for each month using January, the lowest seasonal collection month, as a reference. An initial peak with a 3.9 fold change from January [FCJan] (95% CI: 2.6, 6.1) was observed in May and July (3.8 FCJan, 95% CI 2.4, 6.0). This was followed by a dip in collections in August (2.0 FCJan, 95% CI: 1.2, 3.3) and September (1.3 FCJan, 95% CI: 0.8, 2.2) and a second upward trend peaking in November (3.6 FCJan, 95% CI: 2.4, 5.8) (Figure 2A). By village, we found that 45% (n = 3,654) of all *P. duboscqi* (male and female) were collected in Kemena and 54% (n = 4,276) in Sougoula. Using the quasi-Poisson model, controlling for previous count, month, and year we observed a significant difference in total *P. duboscqi* counts between the two villages (p-value 0.0293) with the sand fly counts 1.188 times higher, on average, in Sougoula than in Kemena (95% CI: 1.025,1.377) (Figure 2A). Similar results hold when using only female sand fly counts (fold-change = 1.155, p = 0.1116, Figure 2B). The various weather variables (relative humidity, rainfall amount, maximum or minimum temperature, and maximum and minimum wind velocity) were not useful for predicting the observed total or female sand fly collections for either village (all models had p-value >0.53) (Figure 3B).

**Figure 4:**
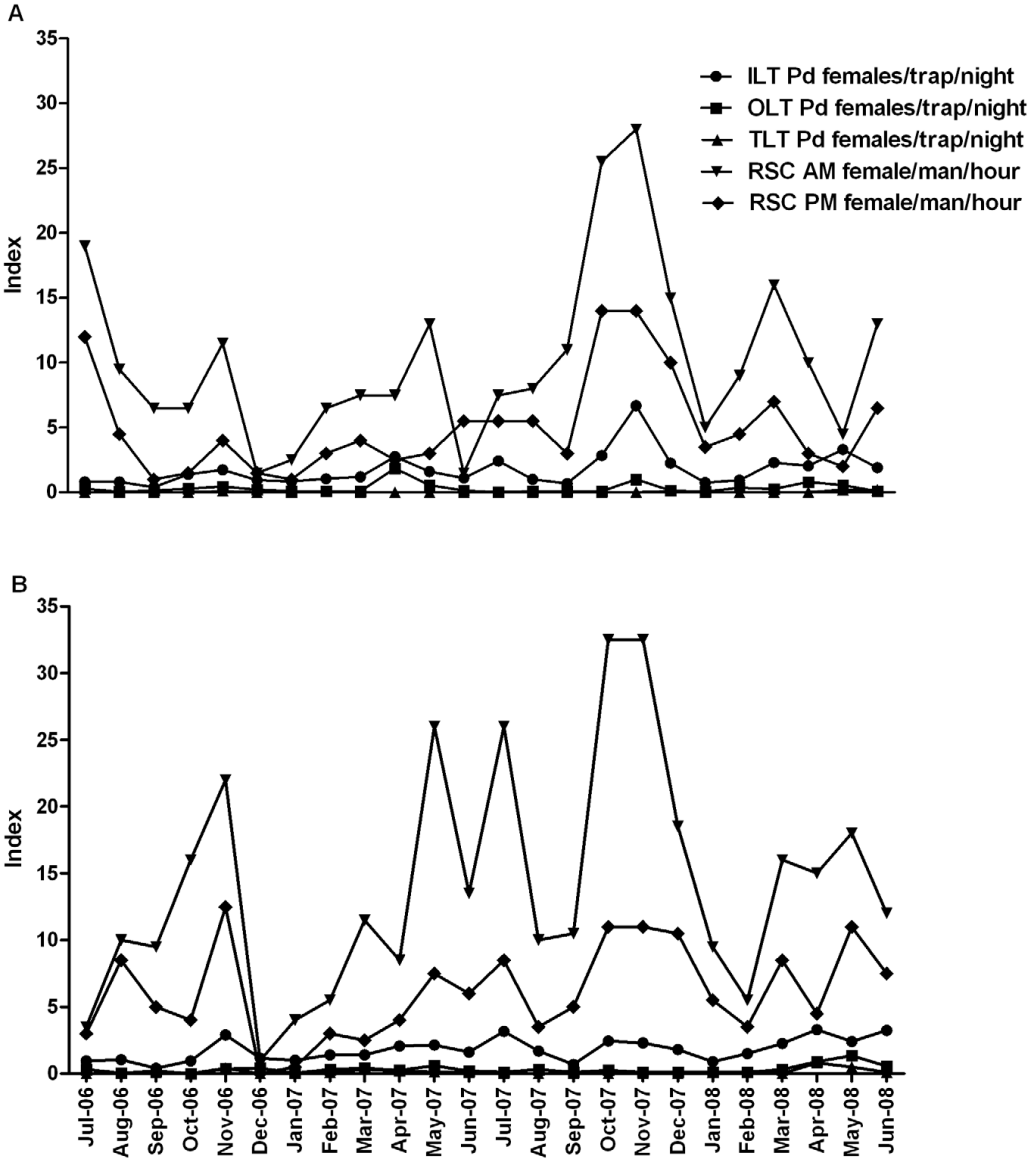
Collection success of female *Phlebotomus duboscqi* using various trapping methods. Number of female *P. duboscqi* collected per trapping method per night during monthly collection from July 2006 to June 2008 in two neighboring villages, Kemena (A) and Sougoula (B), in Central Mali: morning resting site collection (RSC AM)  =  female *P. duboscqi* (Pd) collected during 2 man hours, evening resting site collection (RSC PM)  =  female *P. duboscqi* collected during 2 man hours, tree light traps (TLT)  =  female *P.duboscqi* collected by 10 traps, indoor light traps (ILT)  =  female *P duboscqi* collected by 20 traps, outdoor light traps (OLT)  =  female *P. duboscqi* collected by 20 traps.

The majority of *P. duboscqi* was collected by resting site collection, particularly during the morning (10.54 and 14.04 female *P. duboscqi*/person/hour during morning collections in Kemena and Sougoula, respectively, compared to 5.12 and 6.10 *P. duboscqi*/person/hour during evening collections) (Figure 4). On average, five times more *P. duboscqi* were collected using light traps placed inside of dwellings than outside in the same compound (1.74 vs. 0.33 and 1.78 vs. 0.31 *P. duboscqi* females/trap/night in Kemena and Sougoula, respectively) (Figure 4). Virtually no *P. duboscqi* were collected in the light traps placed outside of the village near natural tree holes (0.03 and 0.12 *P. duboscqi* females/trap/night in Kemena and Sougoula, respectively).

### Infection of *P. duboscqi* with *L. major*

**Figure 5:**
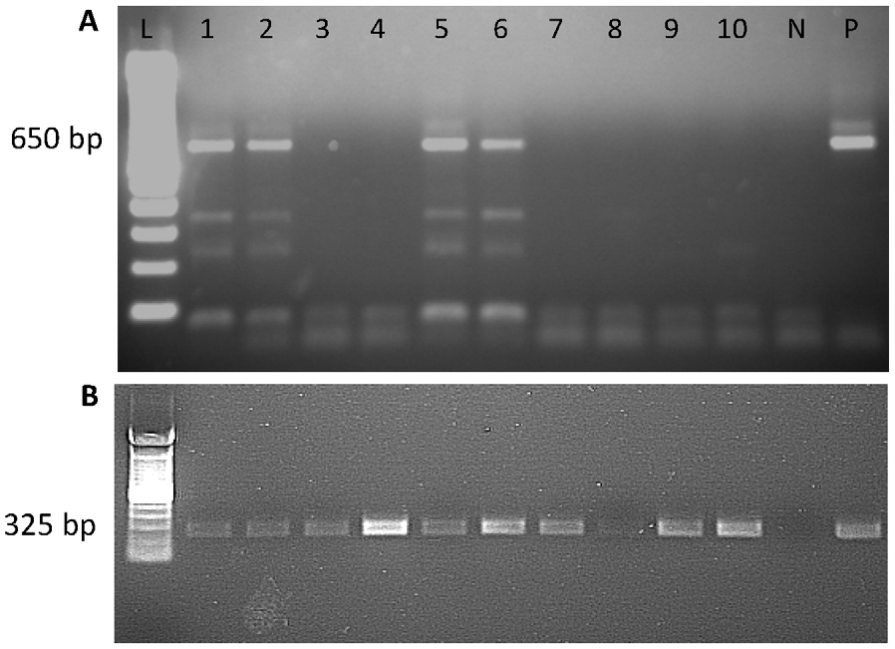
*Leishmania* specific PCR products from field collected female *Phlebotomus duboscqi.* *Leishmania* specific (A) and tubulin loading control (B) PCR products. N  =  negative control, P  =  positive control (DNA from *P. duboscqi* experimentally infected with *L. major*). L  =  100 bp ladder. Predicted band size of the PCR product for (A) *L. major* (650 bp) and (B) tubulin (325 bp) is indicated.

**Figure 6:**
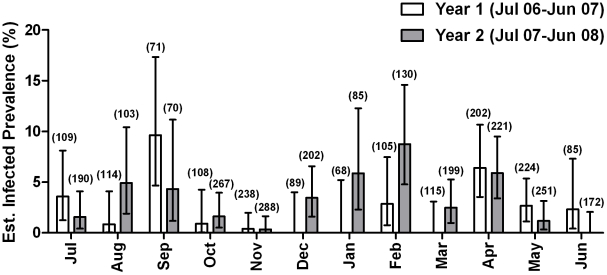
Estimated prevalence of infected sand flies collected from Kemena and Sougoula, two neighboring villages in Central Mali. Numbers in parenthesis represent the overall number of female *P. duboscqi* specimens tested in pools of 1–20 flies.

A total of 1434 pools (3706 total flies; average 2.6 flies per pool) were examined for *Leishmaina* infection by PCR. Ninety-seven pools were positive for *L. major* (Figure 5). Assuming that the sand flies are independently distributed in the pools and the size of the pools is not related to the probability of infection of the pool, we estimate the prevalence of infection to be 2.66%, 95% CI: 2.20, 3.21 (Table 2). Infected *P. duboscqi* were found during each month of the year, although monthly infection estimates varied greatly year to year, being the highest during September 2006 (9.64%; CI: 4.68, 17.34) and February 2008 (9.19%; 95% CI: 5.03, 15.27) (Figure 6). After controlling for sand fly count, there was no significant difference in the rates of infection from year 1 to year 2 (p-value 0.2572) and neither was there a significant month to month difference (p = 0.2085). The estimated infection prevalence of sand flies was virtually the same for both villages (2.65%, 95% CI: 1.97, 3.51 and 2.67, 95% CI: 2.02, 3.44 for Kemena and Sougoula, respectively) with no significant difference between the two villages (p-value 0.8894) (Table 2). Of the sand flies collected by light traps and resting site collections within compounds, the majority of infected sand flies were collected using light traps versus resting site collection (4.14% of vs. 0.86%). The highest estimated prevalence of infected sand flies was collected from compound 5 in Kemena (9.42%; 95% CI: 5.50, 14.92) and compound 1 in Sougoula (5.94%; 95% CI: 2.97, 10.57). Comparing the position of the light traps, the estimated infection prevalence of sand flies collected in light traps placed directly outside dwellings was higher than those placed inside dwellings (8.15% vs. 2.07%); no infected sand flies were collected from light traps placed in trees outside of either village (Table 2). The estimated infection prevalence of flies that were non-blood fed at the time of collection was 4.01% versus 1.24% for those that were blood fed.

**Table 2:** Estimated infection prevalence of *Leishmania major* in female *Phlebotomus duboscqi* from July 2006–June 2008.

	Kemena					Sougoula					Total				
	No. pools	No. Flies	No. Pools Infected	Estimated Infection Prevalence	95% Confidence interval	No. pools	No. flies	No. Pools Infected	Estimated Infection Prevalence	95% Confidence interval	No. pools	No. Flies	No. Pools Infected	Estimated Infection Prevalence	95% Confidence interval
**Total**	674	1670	44	2.65	1.97–3.48	760	2022	53	2.67	2.04–3.44	1434	3706	97	2.66	2.20–3.21
**By collection method**															
LT (inside/outside houses)	487	967	40	4.23	4.23–3.09	537	1076	43	4.05	3.00–5.35	1024	2043	83	4.14	3.34–5.06
RSC	185	715	4	0.56	0.18–1.33	208	925	10	1.10	0.57–1.93	393	1640	14	0.86	0.50–1.41
LT (trees, outside village)	2	2	0	0.00	0–84.18	15	21	0	0.00	1.00–16.11	17	23	0	0.00	0.00–14.82
**By location of collection**															
Interior (LT/RSC)	551	1508	28	1.87	1.28–2.64	625	1832	40	2.23	1.63–2.98	1176	3340	68	2.07	1.63–2.59
Exterior (LT outside of houses)	123	176	16	9.59	5.80–14.83	135	190	13	6.86	3.91–11.10	258	366	29	8.15	5.65–11.33
**By compound**															
1 (LT)	119	282	10	3.64	1.90–6.35	93	155	9	5.94	2.97–10.57	−		−	−	−
2 (LT)	111	215	8	3.79	1.80–7.02	106	201	11	5.59	3.02–9.44	−		−	−	−
3 (LT)	114	232	6	2.59	1.10–5.25	135	302	9	2.99	1.50–5.36	−		−	−	−
4 (LT)	54	85	2	2.35	0.43–7.44	109	216	6	2.80	1.16–5.68	−		−	−	−
5 (LT)	91	155	14	9.42	5.50–14.92	109	223	8	3.59	1.72–6.61	−		−	−	−
6 (RSC)	58	210	2	0.94	0.17–3.00	58	164	3	1.81	0.49–4.75	−		−	−	−
7 (RSC)	72	336	2	0.59	0.11–1.90	77	399	2	0.50	0.09–1.60	−		−	−	−
8 (RSC)	55	169	0	0.00	0–2.12	73	362	5	1.45	0.55–3.18	−		−	−	−
**By blood meal status**															
Bloodfed	265	798	11	1.38	0.74–2.36	290	999	11	1.12	0.60–1.93	555	1797	22	1.24	0.80–1.83
Non-bloodfed	409	886	33	3.81	2.69–5.23	470	1023	42	4.17	3.08–5.52	879	1909	75	4.01	3.20–4.95

Eight representative PCR products from infected wild caught *P. duboscqi* were sequenced using primers specific to the kinetoplast minicircle DNA of *L. major.* Sequence analysis confirmed that all the samples were similar to published *L. major* sequences based on length of the product and primer region identity. Blast analysis indicated a best match to *L. major* kinetoplast DNA (Genbank Accession number Z32842.1, E-value 9e-48). Further alignment of the sequences obtained from this study with known *Leishmania* sequences of other species in Genbank confirmed that the 650 bp fragment size, observed on gel electrophoresis of the PCR products, as characteristic of *L. major* strains.

## Discussion

Killick-Kendrick [31] suggested the following criteria for incrimination of a vector sand fly: proven anthropophilic behavior and isolation and identification from the sand fly of the same species of *Leishmania* that infects man. Further evidence such as the demonstration that the sand fly feeds on the reservoir host (if known), concordance between the geographic distribution of the suspected sand fly and human disease, proof that the parasite develops in the fly and experimental transmission of the parasite by the bite of the fly can reinforce the incrimination. Based on monthly collections of sand flies over three years in two villages in central Mali, where CL is known to be endemic, we have demonstrated that *P. duboscqi* is the predominant *Phlebotomus* species; that it persists throughout the year; that females are primarily collected inside houses in both villages;and that it has an overall infection rate with *L. major* of 2.66% as demonstrated by PCR. This strongly points to *P. duboscqi* as the primary vector of *L. major* in Mali.

Species of the sub-genus *Sergentomyia* constitute the majority of sand flies collected in both villages with *S. schwetzi* being the most abundant. Members of the *Sergentomyia* genus are known to transmit *Sauroleishmania* among lizards. *Sergentomyia schwetzi* is the only *Sergentomyia* species known to be anthropophilic and was considered a possible vector by Parrot [5] in 1943. Later, Lawyer [32] concluded that despite the anthropophilic behavior of *S. schwetzi*, it was not a vector of *Leishmania* in humans. In this study, only 1.6% of all *Sergentomyia* sand flies were found during resting site collections and the overall majority (70%) was collected outside of dwellings, further supporting the exophilic nature of this sub-genus and the improbability that *Sergentomyia* sand flies are involved in transmission to humans in our two villages.

Two *Phlebotomus* species were found during the three collection years, *P. duboscqi* and *P. rodhaini*, both of which are known vectors of *L. major* elsewhere in West Africa. While fewer in number than *Sergentomyia* species, *P. duboscqi* was predominantly collected by resting site collection from sleeping dwellings and five times more *P. duboscqi* females were collected in light traps placed inside than outside of dwellings, supporting the anthropophilic nature of this fly. *P. duboscqi* was collected in similar numbers in both villages year round with two seasonal peaks, May-July and October-November. These results are consistent with Lariviere [11] who reported on the seasonality of 191 *P. duboscqi* collected in Mali. The collection of *P. duboscqi* throughout the year is probably the result of having constant monthly temperatures and a relative humidity that does not drop beyond 18%. However, non of the specific weather parameters tested could be significantly correlated with sand fly collections in either village (Figure 3). Few specimens of *P. rodhaini* were collected throughout the study period indicating that this species probably does not play a role, or plays a minor role, in the transmission of *L. major* in Central Mali.

To further incriminate *P. duboscqi* as the vector of *Leishmania* in our study villages, we tested 3706 specimens (in 1434 pools) for the presence of *Leishmania* DNA by PCR. We found that 97 of the pools tested positive for an overall estimated sand fly infection prevalence of 2.66%. None of the infected pools contained *P. rodhaini*. Since the infection rate in wild-caught sand flies is usually low [31], PCR was used to permit the efficient screening of a large number of specimens. Having established the infection rate of *P. duboscqi* in this region, we plan to isolate a viable culture of *L. major*, necessary to type the strain using traditional methods such as isoenzyme analysis. It is worth noting that in 2006 we established a colony of *P. duboscqi* collected from our two study villages. Subsequently, females from this colony were used successfully to transmit *L. major* to an animal model of CL [33] further supporting the status of this species as a competent vector of CL in Central Mali.

A recent study by our group [19] found that there is an unexplained discrepancy between the prevalence of leishmanin skin test (LST) positivity in our two study villages, Kemena (45% LST positive) and Sougoula (20% LST positive), despite the fact that the villages are geographically and demographically similar and are only 5 km apart. Furthermore, this discrepancy was consistent over two consecutive annual incidences (18% and 17% in Kemena vs. 5.7% in both years in Sougoula). We hypothesized that the sand fly density and infection prevalence may explain the dissimilar LST results. The two year seasonality study revealed that slightly more female *P. duboscqi* were collected in Sougoula than in Kemena, yet almost the same percentage of pools were infected in each village (2.67% vs. 2.66%, respectively), thus neither abundance nor infection prevalence can explain the disparate LST rates observed in the two villages [19].

Rodent species are well known reservoirs for *L. major* throughout its distribution range. The contribution of reservoirs to the observed disparity of LST positivity in the two villages remains to be evaluated. In West Africa, including Senegal where *P. duboscqi* has been incriminated as the vector of *L. major*, infected *Mastomys erythroleucus*, *Tatera gambiana* and *Arvicanthis niloticus* have been reported [34], [35], [36], [37]. All three species are found in Mali (T. Schwan, personal communications) and represent potential reservoirs of *L. major* in Kemena and Sougoula. Indeed, rodent burrows were observed in many of the houses where light traps were placed. Apart from the potential role of these rodents as reservoirs, their burrows also represent suitable sand fly breeding sites and a source of infected flies. Furthermore, all compounds in our study villages contain goats and chickens living in close proximity to houses used for sleeping which also represent good sand fly breeding sites for uninfected flies. A comprehensive study of the rodent population density and infection prevalence in the two villages is needed to fully understand the infection dynamics in both flies and people.

In summary, we have established, for the first time, the diversity of sand flies in two villages endemic for *L. major* in Central Mali and demonstrated by PCR that *P. duboscqi* is the primary vector. This work represents the most comprehensive analysis of *P. duboscqi*, to date, in Mali and further supports the endemic nature of CL in Central Mali. Further investigations of this nature are needed in West Africa.
